# The high-throughput atomization of polymer solutions for fiber synthesis in a single step aided with corona ionizers

**DOI:** 10.1038/s41598-023-39801-3

**Published:** 2023-08-03

**Authors:** Luis B. Modesto-López, Alfonso M. Gañán-Calvo

**Affiliations:** 1 Departamento de Ingeniería Aeroespacial y Mecánica de Fluidos, ETSI, Universidad de Sevilla, Camino de los Descubrimientos S/N, 41092 Seville, Spain; 2 ENGREEN, Laboratory of Engineering for Energy and Environmental Sustainability, Universidad de Sevilla, 41092 Seville, Spain

**Keywords:** Fluid dynamics, Engineering

## Abstract

Polymer microfibers are ubiquitous structures across virtually all technological fields. Their applications include, for instance, filter media, tissue regeneration, wound healing and dressing, and reinforcement materials. The most effective methods for fabrication of fibrous micro and nanomaterials rely on electric fields to spin a liquid jet into an ultrafine thread that rapidly dries up forming a fiber. Continuous spinning and collection leads to formation of fiber mats. Here we report a robust yet simple approach for the massive production of liquid threads, which upon acquiring electrical charges in-flight are collected downstream in the form of fibers. The entire process takes place on-line in a single step. The liquid threads are produced through the fragmentation of a polymer solution bulk due to a turbulent interaction of a gas–liquid interface in the interior of an engineered device, a so-called Flow Blurring atomizer. The particularity of this approach consists precisely in such vigorous interaction, at the micrometer scale, which triggers a bubbly motion in the interior of the device, that is a “micro-mixing”. Subsequently, the threads are passed through ionized air currents, at ambient conditions, and then stretched to sub-micrometer dimensions by electric fields. Polyvinylpyrrolidone (PVP) as well as carbon nanotubes (CNTs) or graphene oxide sheets (GOSs)-containing PVP fibers, with diameters in the range 100–900 nm, were synthesized via this approach. In the cases studied herein the method was operated at liquid flow rates (i.e. production rates) of 0.2 mL/min but it could be readily increased up to a few tens of mL/min. The method requires further improvement and optimization, nevertheless it is a promising alternative for mass production of polymer fibers.

## Introduction

Polymers are versatile macromolecules with an immense spectrum of industrial and technological applications. Their presence in virtually every field of science and engineering has advanced in parallel with the development of processing techniques^1–5^. Most of these procedures involve the preparation of solutions that can be dispensed or transported towards a desired target^1^. To that end, one of the most frequent approaches is liquid atomization, that is, the fragmentation of a liquid bulk followed by its disintegration into drops due to the action of mechanical and/or electrical forces using an engineered device, an atomizer^6–12^. One of the preferred forms of liquid atomization is pneumatic atomization, which is known for its high volumetric throughput and the availability of a wide variety of such type of atomizers. Gañán-Calvo developed an effective form of pneumatic atomization called Flow Blurring (FB), in which the mechanical energy carried by a gas current is efficiently used to break up a liquid flow and create new surface^11,12^.

**Figure 1:**
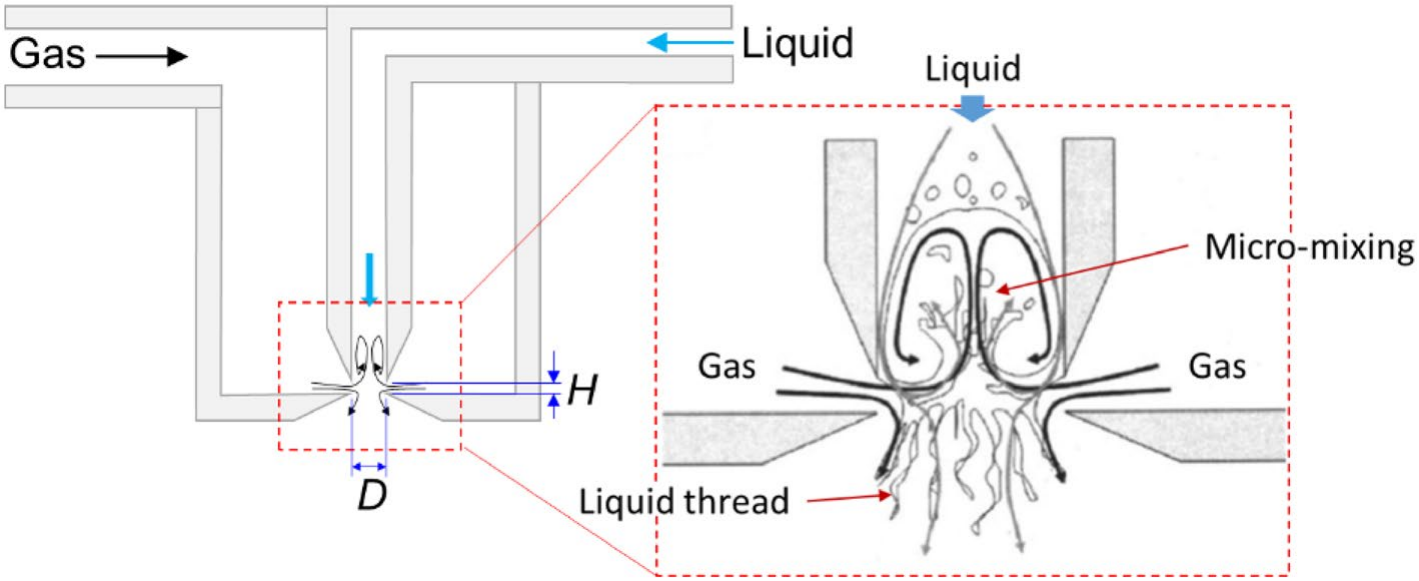
Cross-sectional diagram of a micro-mixing device. $H=250 \; \upmu \text{m}$, $D=1200\; \upmu \text{m}$.

A FB atomizer consists of a system of two concentric capillary tubes that are joined downstream at the tip end, near the discharge zone. In a FB atomizer, the gas stream, flowing through the external capillary, undergoes radial implosion into the liquid feeding tube, thereby inducing a vigorous turbulent motion that triggers micro-mixing within the device (Fig. 1). This mechanism leads to the disintegration of the bulk into tiny droplets. In FB atomization, factors such as liquid flow rate, viscosity, and surface tension determine the size of the ejecta^13^. In addition, the internal geometry of the atomizer (Fig. 1), which is determined by the distance from the tip of the inner capillary to the discharge orifice ($H$) and the discharge orifice diameter ($D$), plays a key role in the liquid breakup. For instance, if the relationship $\phi =H/D$ $<0.25$ is met, the vigorous micro-mixing takes place, conversely for $\phi >0.25$ the gas current functions as focuser and the so-called Flow Focusing occurs^11,12^. The physical characteristics of the FB atomizer and their relationship with operational parameters were described by us and others^11,14–17^. This atomization method has primarily been employed for generating aerosols or micro/nanoparticles from Newtonian liquids^10,11,15,18–20^.

However, we have recently developed a novel application for FB atomization that involves the production of filaments from non-Newtonian fluids, specifically polymeric solutions. This application is enabled by harnessing the micro-mixing mechanism's capability to fragment viscous liquids. In such cases, FB generates multiple liquid threads that retain their shape throughout their displacement^21,22^. These filamentary structures can then be utilized as molds or carriers for synthesizing micro or nanofibers. Hereafter, we use the terms filament and liquid thread interchangeably. One of the advantages of this approach lies in its ability to operate steadily at relatively high liquid flow rates, resulting in high production rates ranging from $0.1 \; \mathrm{mL}/\mathrm{min}$ up to $2 \; \mathrm{L}/\mathrm{min}$, depending on the atomizer's geometry^11,15,16,20–26^.

Here, we introduce an innovative concept that combines FB atomizers as efficient liquid dispersers, generating multiple filaments, with in-flight ionization technology for the production of polymer micro- and nanofibers. That is, as the threads of polymeric solutions are ejected out of the atomizer, by solely pneumatic means, they are subsequently charged downstream as they move forward towards a collector. One of the key advantages of this technique is its ability to achieve mass production of fibers in a continuous and single-step process. Unlike electrohydrodynamic approaches, which require electrically conductive liquids for ejection to take place^27^, our approach utilizes ion collisions during filament displacement for charging, eliminating the need for electrically conductive liquids (or at least substantially reducing the dependence on the electrical conductivity of the liquid, an issue whose detailed analysis beyond this work is currently under study). Furthermore, in this work, a particular configuration is presented in which the as-ejected threads do not pass through the ionizer, but instead, they go through an ionized air current. Thus, eventual accumulation of liquid on the ionizers’ electrodes is prevented which allows a smooth and continuous operation of the system, resulting in an improvement of the work of Modesto-López and Olmedo-Pradas^28^.

With careful design and optimization, the concept presented here holds potential for high-throughput material production. To demonstrate the feasibility of the technique, we propose the use of polyvinylpyrrolidone (PVP) as a test polymer. PVP offers numerous cutting-edge applications due to its unique properties, including its ability to bind to various molecules such as dyes, metals, and other polymers. Furthermore, PVP exhibits low toxicity and high biocompatibility with living tissue, making it an ideal carrier for hydrophilic or hydrophobic drugs and for DNA encapsulation^29^. Crosslinked forms this polymer are used as disintegrants in pharmaceutical tablets^30^. Beyond pharmaceutical applications, PVP finds utility in diverse areas, for instance, PVP fiber mats have applications as membranes for air filtration, oil–water emulsion separation^31,32^, supercapacitors^33,34^, antibacterial wound dressings^35^, flexible rewritable media^36^, and hemostatic bandages^37^. The versatility of PVP makes it a promising candidate for exploring the potential of our novel fiber production approach.

## Experimental section

### Materials

**Figure 2:**
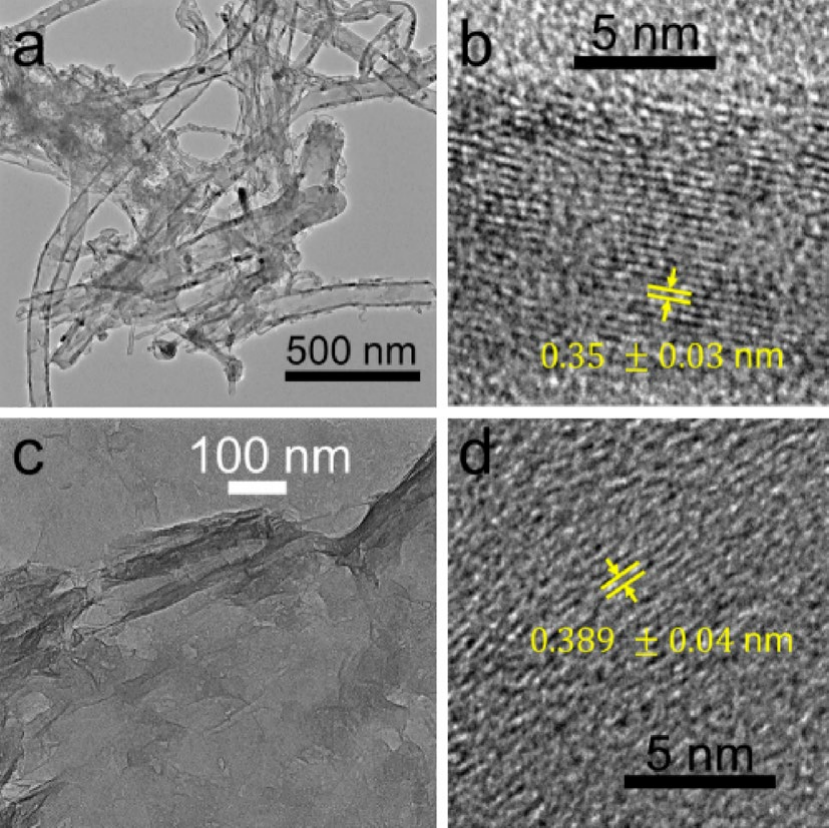
TEM images of CNTs (**a** and **b**) and GOSs (**c** and **d**).

Polyvinylpyrrolidone (PVP) of molecular weight, ${M}_{w}$ = 1,300,000 g/mol was purchased from Merck and used as received. In this study, a relatively high value of ${M}_{w}$ is preferred to ensure that filaments are ejected (instead of droplets)^13,21,22,38^. Aqueous solutions of multilayered graphene oxide sheets (GOSs) sheets and multi-walled carbon nanotubes (CNTs) of 0.2 wt% and 4.15 wt%, respectively, were purchased from Grupo Antolín (Spain) and used as received. Figure 2 shows transmission electron microscopy (TEM) images of as-received CNTs and GO sheets. Ethanol (PanReac, 99.5% v/v, CAS: 64-17-5) was used as solvent, and compressed air was used as the atomizing gas.

### Preparation of solutions

To prepare polymer solutions, a weighted amount of PVP was added to a given volume of ethanol and mixed under mild stirring with a magnetic stirrer until a clear solution was obtained. The weights of PVP and ethanol were such that the final solution concentration amounts 12 wt%. To prepare solutions containing GOSs and CNTs, after PVP solution preparation, $5\; \mathrm{mL}$ of CNTs or GOSs aqueous suspensions were added for every $100\; \mathrm{mL}$ of polymer solution, giving a total PVP concentration of 12 wt%. The weight of water contained in the suspensions was considered for calculations of concentration. The final concentration of CNTs and GOSs were approximately $0.002 \;\mathrm{wt}\%$ and $0.001 \;\mathrm{wt}\%$, respectively. Solutions of ethyl cellulose (EC, average ${M}_{w}= 220{,}000\; \mathrm{g}/\mathrm{mol}$) of varying concentrations were prepared to carry out ancillary viscosity measurements. In addition, viscosity data of poly(vinyl alcohol) [PVA] and poly(ethylene glycol) [PEO] were obtained from previous literature reports^13,21,22,38^.

### Characterization of polymer solutions

The densities of the PVP solutions (${\rho }_{l}$) were obtained by weighting a known volume of solution. Surface tension ($\sigma$) values of polymeric solutions in air were measured with a KSV Instruments’ contact angle meter (model CAM100) set up in a pendant drop configuration for static measurements in the range 0°–180°. The instrument is equipped with a FireWire video camera module with a resolution of $640 \times 480$ pixels and with a light-emitting diode, monochromatic, light source. The objective lens provided with the camera is telecentric with a 55 mm focal length. The instrument’s software applies curve fitting using the Young–Laplace equation to calculate surface tension. The solutions’ shear viscosities were obtained using a TA Instruments rheometer (Discovery HR-3) with cone–plate configuration with a gap of 52 µm. The cone angle and the plate diameter were $2.009^\circ$ and 60 mm, respectively. Temperature control was achieved using a Peltier plate equipped in the instrument. The zero-shear viscosity value, ${\mu }_{0}$, was taken as the viscosity at the lowest shear rate (0.1 s^−1^) and was 0.5 Pa s for the PVP-only solution and 0.6 Pa s for that containing CNT or GO. The three solutions exhibited a Newtonian behavior up to approximately 20 s^−1^, where the viscosity began to slowly decrease with shear rate. The viscosity measurements were performed at room temperature and ambient relative humidity (in the range of 50–60%). The specific viscosity ${\mu }_{sp}$ (dimensionless) of solutions was calculated as $({\mu }_{0}-{\mu }_{s})/{\mu }_{s}$, where ${\mu }_{s}$ is the solvent’s Newtonian viscosity at room temperature. Table 1 summarizes the results of the characterization of PVP solutions.

**Table 1:** Experimental conditions for fiber production with a PVP solution of 12 wt%.

Entry	${\rho }_{l}$ [kg/m^3^]	${\mu }_{0}$ [Pa s]	$\sigma$[N/m]	$\Delta {P}_{g}$[kPa]	${Q}_{g}$ [L/min]	${Q}_{l}$ [mL/h]	${\overline{d}}_{f}$ [nm]	Additives
1	816	0.5	0.0213	81.2	15	2.04	934	
2				81.2	15	2.40	904	
3				81.2	15	6.60	813	
4				81.2	15	11.7	611	
5	856	0.6	0.0264	82.0	15	47.1	986	CNTs
6	862	0.6	0.0233	82.0	15	14.8	994	GOSs

### The flow blurring atomizer

The micro-mixing-based Flow Blurring (FB) atomizer was obtained from Ingeniatrics Tecnologías S. L. (Sevilla, Spain). As mentioned earlier, FB atomization devices are characterized by the dimensionless parameter $\phi =H/D$ (refer to Fig. 1), where $H$ represents the distance from the tip of the inner tube to the discharge orifice, and $D$ is the diameter of the discharge orifice. In our experiments, we utilized a device with $\phi =0.21$, which demonstrated stable operation at high liquid flow rates, reaching up to a few liters per minute. This allowed for a continuous and consistent liquid ejection. Furthermore, the relatively larger size of our device and its larger gas passage clearance (compared to a device with $\phi =0.14$ as utilized by Ramos-Escobar et al.^13^) reduces the risk of clogging.

### Development and functioning of ionization devices

**Figure 3:**
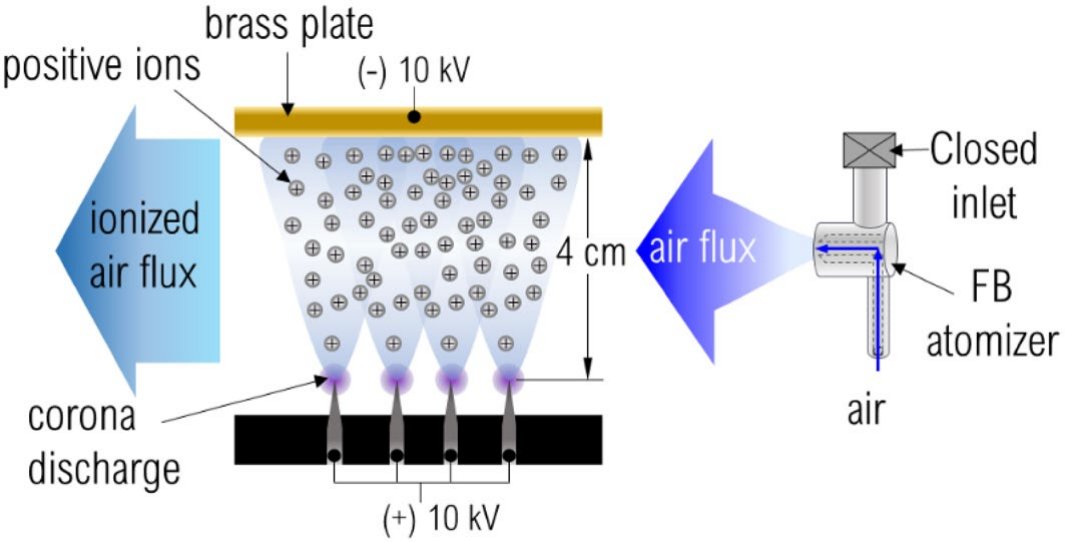
Sketch of ionization system.

**Figure 4:**
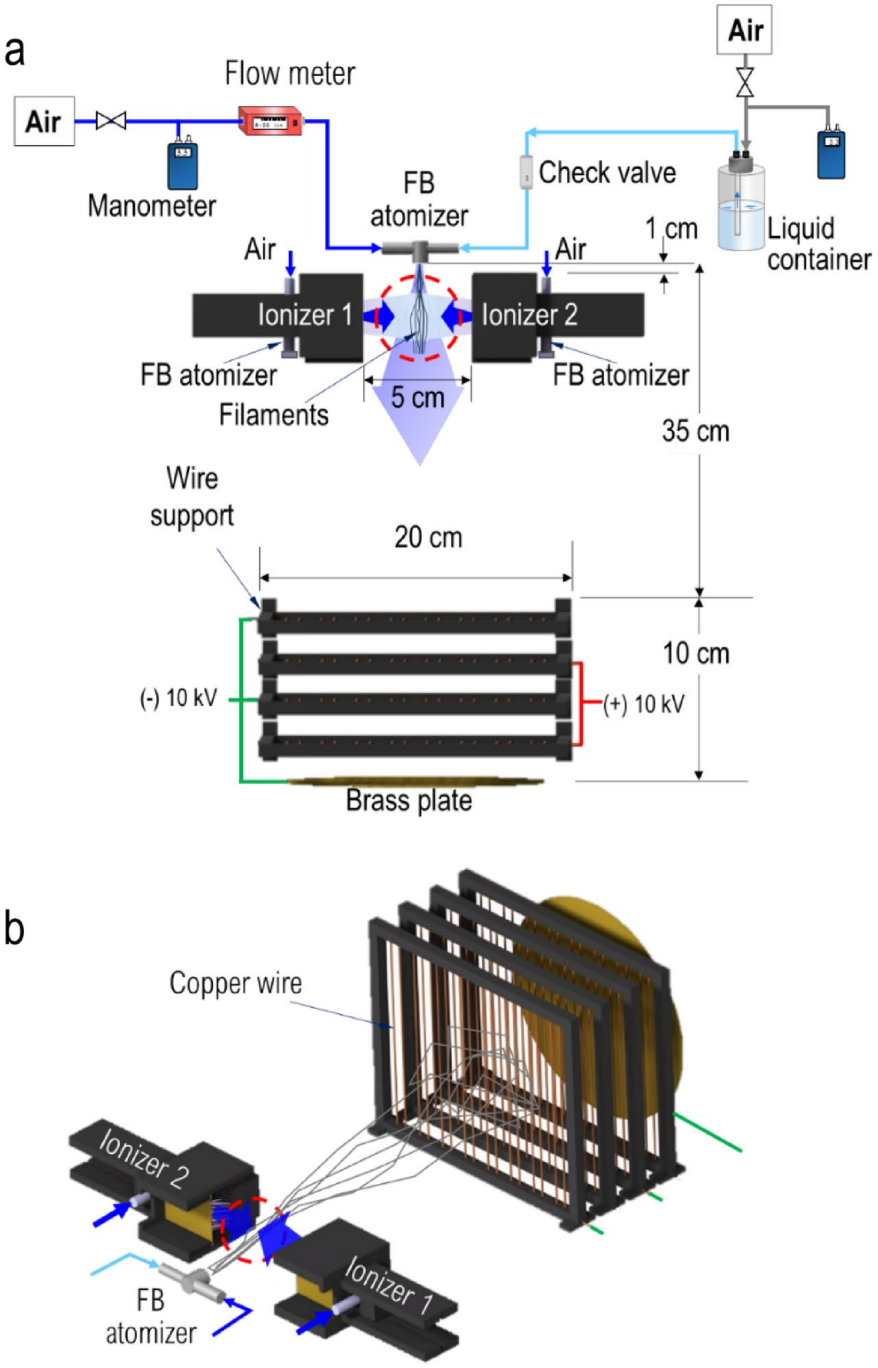
Experimental setup for micro- and nanofiber fabrication. (**a**) Top view and (**b**) Perspective view.

Two ionizers were used for electrically charging air flows which then would mix up with as-ejected liquid threads. The ionizers operate at ambient temperature and pressure conditions based on the corona discharge principle. Their bodies were fabricated by 3D printing using standard PLA filament according to an in-house design, which was described by Modesto-López and Olmedo-Pradas^28^. The schematics of Fig. 3 illustrates the process of ions’ generation. Briefly, each ionizer consisted of a needle-to-plate configuration where $+10\; \mathrm{kV}$ were applied to the needles and $-10 \;\mathrm{kV}$ to a brass plate thus generating currents of approximately 1 mA per ionizer unit. The needle-to-plate distance was 4 cm. In the current configuration, the as-generated ions were transported out of the ionizer by means of pressurized air discharged from a FB atomizer (Ingeniatrics Tecnologías S. L., Sevilla, Spain), which was placed in the interior of the ionizer body. In this case, the atomizer had the sole purpose of supplying air at flow rate of $30 \;\mathrm{L}/\mathrm{min}$ to carry away ions (no liquid is involved). The ionizers were placed in a front-to-front configuration so that the ionized air currents coming out of them slam into each other thus creating a zone with high ion concentration (Fig. 4).

FB atomizers, identical to the one used for atomization of polymer solutions, were employed to ensure the generation of identical turbulent spectra for the gas streams. This approach aimed to maximize the turbulent contact between the ion fluxes carried by the gas streams and the smaller turbulent scales originating from the atomized solution stream, thus achieving optimal rate and efficiency.

### Micro-mixing atomization of polymer solutions and fiber collection

Figure 4 illustrates the experimental setup used to produce fibers of PVP, CNTs-PVP, and GOSs-PVP. The experimental procedure involved delivering air into the device at a fixed gas flow rate (${Q}_{g}$) of $15 \; \mathrm{L}/\mathrm{min}$ measured with a digital flow meter (Red-y compact series, Vögtlin Instruments), followed by introduction of the polymer solution. The solution was pneumatically fed through a pressurized container, where the overpressure ($\Delta {P}_{l}$) was varied between 70 and 85 kPa, resulting in liquid flow rates (${Q}_{l}$) ranging from 2 to 12 mL/h. A check valve was installed between the liquid container and the FB atomizer to prevent back flow. ${Q}_{l}$ data were obtained through separate experiments by measuring the volume of ejected liquid in a period of time and then establishing a calibration curve. The implosion of the air stream into the liquid feeding tube caused the fragmentation of the bulk liquid and the ejection of filaments^11,14,18^. While FB atomizers are capable of operating at liquid flow rates (production rates) of up to tens of L/h (depending on their geometry)^15,26^, for this study, we employed the lower end of the device's operating liquid flow rates due to laboratory-scale constraints. However, the lower end values of liquid flow ($< 5 \; \mathrm{mL}/\mathrm{h}$) utilized in this work, using a single device, are similar to those commonly used in standard methods or large-scale fiber production techniques^39,40^, while the upper end values of ${Q}_{l}$ (> 10 mL/h) are unparalleled.

The as-ejected filaments were directed through the ionization zone and subsequently towards the collection system, which comprised four sets of electrodes and a brass plate. The four collectors were positioned at a distance of 35 cm downstream of the ejection point to create an electric field. Each collector consisted of a plastic frame with 16 vertically aligned copper wires (1 mm in diameter) spaced 1 cm apart. The collectors were placed sequentially, interleaving the voltage polarity applied to each one: The first collector was connected to a negative voltage, the second to a positive voltage, and so forth. Downstream of the last collector, the circular brass plate was connected to a negative voltage. An electrical potential of ± 10 kV was applied to the collectors and the plate.

In the case of solutions containing CNTs or GOSs, employing similar micro-mixing conditions used for PVP-only solutions did not yield fibers; instead, large droplets were ejected. However, by increasing the temperature of the surroundings using a heat gun, fiber production was facilitated, allowing for operation at higher liquid flow rates with CNT- and GOS-containing solutions. The possible modification of the physicochemical properties of these solutions (e.g. non-Newtonian behavior) and their ability to acquire charges is currently under analysis. In such cases, the heat gun was positioned above the FB atomization device, inclined and pointing towards the ejected filaments. The temperature measured 290 °C and 60 °C at distances of 1.5 and 20 cm from the heat source, respectively.

### Algorithm-based processing of SEM images and characterization of fiber diameters

An in-house image segmentation algorithm was developed, utilizing the widely used engineering program MATLAB^®^ (by MathWorks Inc.), which offers a user-friendly interface. This algorithm enables detailed (pixel by pixel) and rapid processing of SEM images of the fibers, allowing for the quick determination of their diameter distributions. For image processing, an optional "soft processing" step is available, involving adjustments to sharpness and contrast, which also allows for selecting in-focus fibers. The soft processing, combined with image cropping to retain only regions of interest, aims to enhance the algorithm's performance and reduce noise sources.

Subsequently, the SEM images were binarized, representing in-focus fibers as white pixels and background/blurred fibers as black pixels. This binarization process is a type of image segmentation, which facilitates the characterization of the fibers by creating distinct structures. The image binarization was accomplished using the Otsu method^41^, which employs a grayscale intensity threshold to minimize the variance within each category and maximize the variance between the two categories. Detailed information about this method can be found in the work of Zhang et al.^41^.

**Figure 5:**
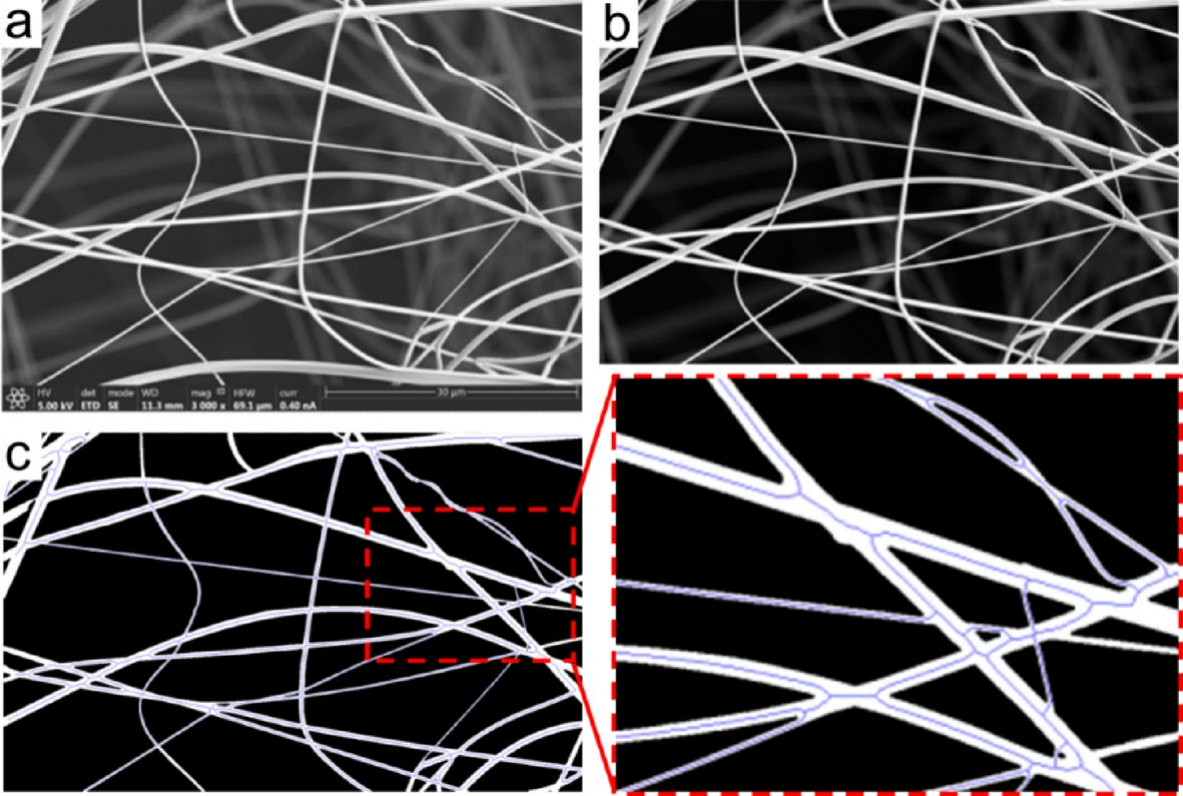
Processing of microscopy images. (**a**) Original image, (**b**) soft-processed image, (**c**) segmented and skeletonized image, where the blue lines represent the fibers’ skeleton.

Following binarization, filtering techniques were applied to suppress noise. In particular, a median filter was used to address the suppression of isolated pixels (white or black), transforming their color to that of neighboring pixels. Subsequently, the fibers' skeleton was computed using the built-in MATLAB^®^ function "*bwskel*", which reduces the structures in the binary image to one-pixel wide curved lines, preserving the topology and Eulerian characteristics of the objects in the image. Finally, the fibers' radii were measured by calculating the distance between a pixel in the skeleton and the nearest pixel belonging to the background of the binarized image. This approach enables multiple measurements to be obtained from a single SEM image, which are used to calculate the diameter distributions. Figure 5 illustrates the process with three images: the original image, the image after soft processing, and the segmented and skeletonized image.

It is important to note that all the information present in an SEM image is utilized to acquire relevant data regarding the fibers. This method not only allows for the calculation of the diameter distribution but also provides insights into the fibers' topology, including porous structures, curved fibers, protuberances, straight fibers, etc. By calculating diameters in a pixel-by-pixel manner, a few thousand measurements can be obtained from each image of a single sample. Combining data from various images of a sample can yield more than 50,000 readily recorded measurements, making the diameter distribution a representative characterization of the sample. Moreover, employing this method for image processing takes a couple of minutes, which is significantly more practical than manual approaches. The associated error term for the measurements obtained using this in-house method is on the order of the distance equivalent to one pixel (in our case, ~ 20 nm). Therefore, higher image resolutions lead to increased precision in the measurements.

## Results and discussion

**Figure 6:**
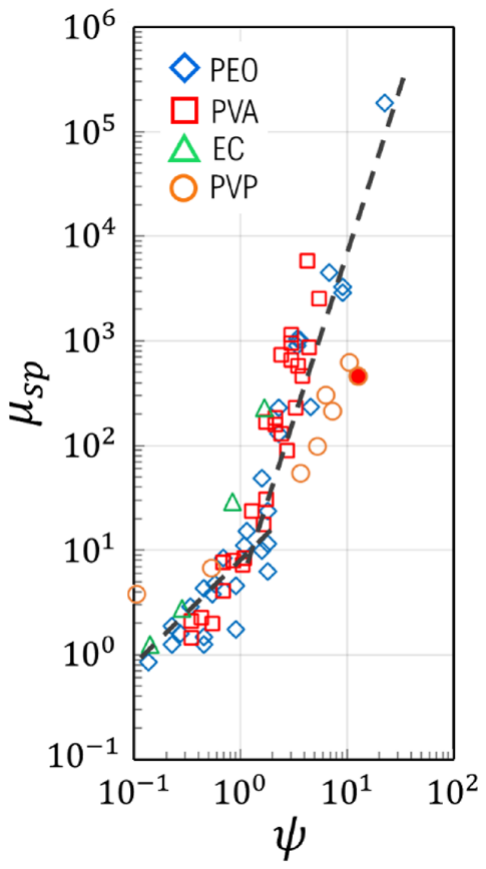
Specific viscosity (zero shear) of polymer solutions of varying molecular weight and concentration. The full, red circle corresponds to PVP of 12 wt% in ethanol. The linear relationship expected for $\psi <1$ undergoes a drastic change as $\psi >1$ (power law fitting suggested as an eye guide only, with no theoretical support).

Solution concentration has a significant impact on viscosity, a crucial factor in the formation of filaments and fibers using the micro-mixing mechanism. Solutions with lower viscosity tend to disintegrate into droplets, while those with higher viscosity tend to elongate and form filaments^21,22^. This behavior is determined by the non-dimensional parameter $\psi$, defined as $\psi =c {M}_{w}/{M}_{e}$, where c represents the mass fraction of polymer in the solution, ${M}_{w}$ is the molecular weight of the polymer, and ${M}_{e}$ is the entanglement molecular weight of the polymer. The critical value of $\psi$, approximately unity, serves as a threshold when plotted with a suitable parameter, in this case, the nondimensional, specific viscosity, μ_sp_: solutions with $\psi <1$ form droplets upon micro-mixing ejection, whereas solutions with $\psi >1$ form filaments that can be further processed into fibers with appropriate treatment. Figure 6 provides a graph of plotted as a function of $\psi$ for different polymers (PEO, PVA, EC, and PVP) with varying molecular weights and concentrations. The graph reveals two distinct regions with different slopes, divided by the critical value $\psi \sim 1$. The change in slope reflects the different rheological behavior of the solutions and is consistent with previous findings supported by others^42–46^. In our work, the PVP solution with a concentration of 12 wt% in ethanol corresponds to a $\psi$ value of 12.7 and a ${\mu }_{sp}=465$, ensuring the ejection of filaments (depicted as a full red circle in Fig. 6).

The volume of solution expelled because of the micro-mixing mechanism through the FB device is generally significantly large, surpassing standard methods by at least two orders of magnitude, and the ejection velocity is approximately 100 m/s. These conditions imply that, at the laboratory scales, there is only a brief time window of a few milliseconds available to transform the filaments into solid material before they collide with the collector. During this time, the filaments face the possibility of losing their thin, one-dimensional structure due to coalescence or other mechanisms^13^. Common techniques often employ electric fields as an external energy source to elongate the filaments and significantly reduce their diameter, facilitating solvent evaporation and solute solidification^47–50^.

**Figure 7:**
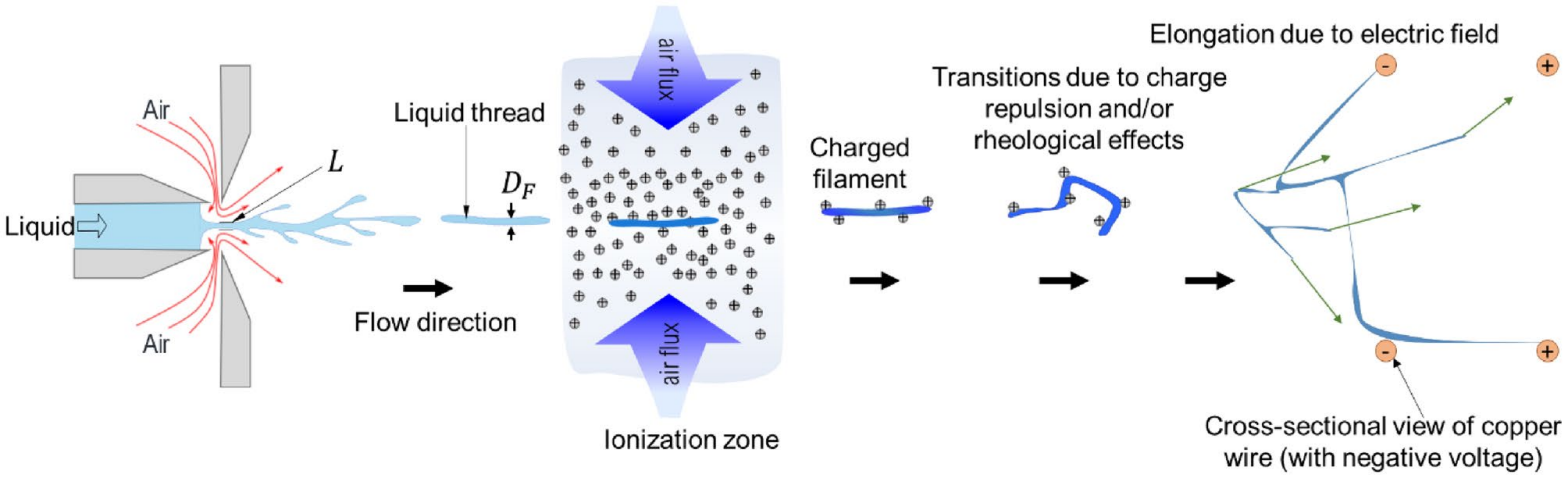
Description of the processes of filament ejection and charging and fiber formation. Green arrows indicate random movements.

In our approach, solutions were pneumatically ejected without any means of electrically charging the bulk solution. By doing so, the requirement for the liquid to be electrically conductive is eliminated. Furthermore, by generating multiple filaments during ejection, the production rate is increased using a single atomizing device. Taking advantage of the generation of multiple liquid threads, we utilize in-flight ionization of the filaments, specifically during their trajectory from the ejection point to the collector. This allows for manipulation of their path using electric fields. Figure 7 qualitatively illustrates the process, showing a liquid thread being expelled towards a zone with a high concentration of ions. The ionization zone is created by two opposing air currents supplied through the ionizers and flowing perpendicular to the main axis of liquid ejection. The flow of ionized air streams with identical turbulent air flow spectra as that of the solution’s nebulizer is continuous, maintaining a steady state with a relatively constant ion concentration. As the filaments pass through this zone, they are assumed to entrain ions on their surface by following an optimally fitted turbulent cascade down to the Kolmogorov microscale. In the in-house designed ionizers, the potential difference between the needles and the plate is 20 kV, and the emitted currents are approximately 1 mA, resulting in a total energy rate of about 20 W per ionizer for the ionized air flow on the liquid threads. With this energy input, the filaments continue their motion towards the downstream collectors. Along this path, they undergo several mechanisms that can modify their morphology until they reach the collectors with a high electric field, where they rapidly elongate, forming thinner fibers. Note that atomization experiments without ionizers did not form fibers but instead the liquid impacted on the electrodes and accumulated there.

In a previous work, we developed scaling laws to predict characteristic length scales and velocities of as-ejected filaments with this micro-mixing approach^13^. One such critical parameter is the average diameter of the ejecta (liquid threads) downstream the ejection point, ${D}_{F}$, depicted in Fig. 7. It is reasonable to think that ${D}_{F}$ will affect the fiber diameter (${d}_{f}$) because these threads become fibers’ precursors. In the current experimental conditions, ${D}_{F}$ is in turn influenced by the characteristic width of the liquid stream in the vicinity of the atomizer’s discharge region, $L$ (see Fig. 7), given by^13^:

$$L\sim {\left({Q}_{l}/U\right)}^\frac{1}{2},$$

where, $U$ is a characteristic velocity of the liquid in the interior of the atomization device and is calculated with ${\left(\Delta {P}_{g}/{\rho }_{l}\right)}^{1/2}$^13^, and for the experimental conditions of Table 1 equals 9.98 m/s. Furthermore, in the cases presented herein typical Reynolds numbers, $Re={\left({\rho }_{l}\Delta {P}_{g}{D}^{2}/{\mu }_{l}^{2}\right)}^{1/2}$, are of the order of 20 and Bond numbers, $Bo={\rho }_{l}g{D}^{2}/\sigma$, are below unity so gravity effects are negligible. Under such conditions, $L$ ranges from 7.5 to 18 µm and in cases, as in this work, where the liquid flow rate or viscosity are sufficiently high to prevent the formation of a relatively large gas cavity at the end of the liquid feeding tube, ${D}_{F}$ is calculated with^13^:

$${D}_{F}\sim {\left({\rho }_{g}^{2}{U}^{3}{Q}_{l}/{\sigma }^{2}\right)}^{1/5}\left(\sigma /{\rho }_{g}{U}^{2}\right){Re}^{-1/5},$$

**Figure 8:**
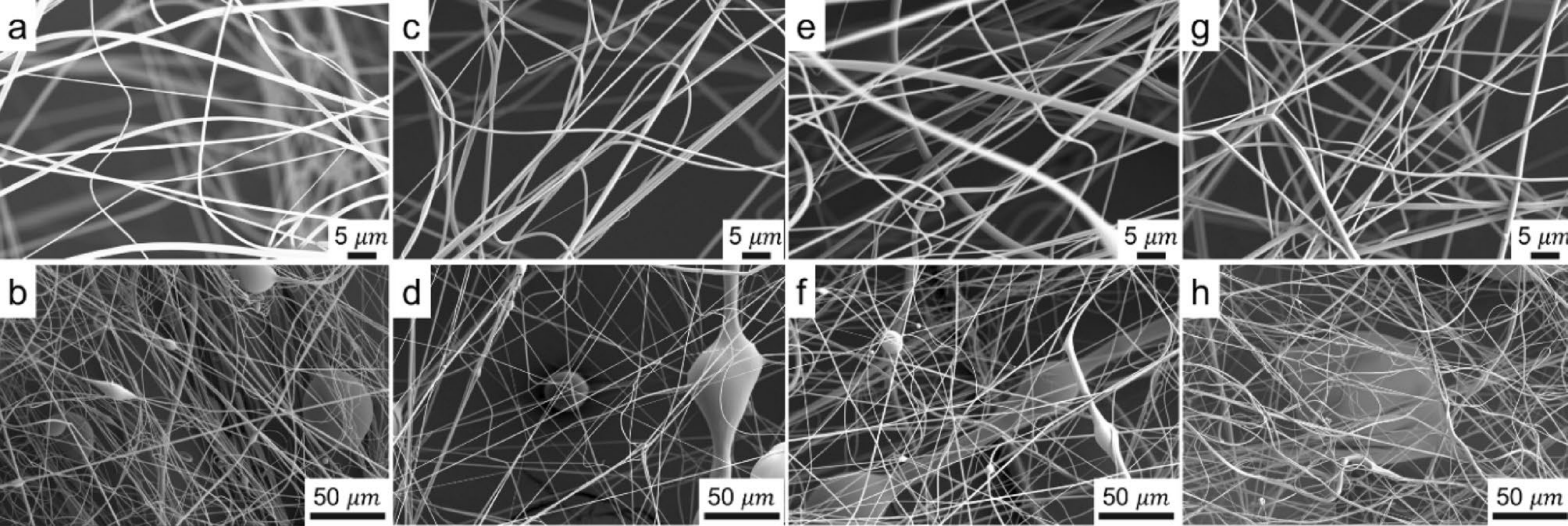
SEM images of PVP fibers obtained under varying liquid flow rates: $2.04\;\mathrm{ mL}/\mathrm{h}$ (**a**,**b**), $2.40\; \mathrm{mL}/\mathrm{h}$ (**c**,**d**), $6.60\; \mathrm{mL}/\mathrm{h}$ (**e**,**f**), and $11.7\; \mathrm{mL}/\mathrm{h}$ (**g**,**h**).

Accordingly, the ${D}_{F}$ values of our experimental conditions are in the range 27–46 µm. This result implies that in order to form fibers with diameter in the submicrometer or nanometer regimes the liquid threads should be subjected to high elongation rates because the entire synthesis process takes place in a relatively short distance and time. Figure 8 shows SEM photograms of PVP fibers fabricated with experimental conditions summarized in Table 1. Here, it is noteworthy to highlight the efficacy of this method in transforming the threads ejected at high flow rates and speeds into solid fibers within a distance of ~ 30 cm from the ionization zone. The synthesized materials were composed of fibers with sizes well below 1 µm but also of thick relics. In addition to those, relics resembling charged droplets with protrusions from so-called Taylor cone ejections are also observed in the images. They are similar to relics formed during the electrospraying or electrospinning of polymer solutions^51,52^, which may indicate the charging of the liquid above the so-called Rayleigh limit^53^.

The results suggest that while some of the liquid threads may transition directly to fibers, others would coalesce and may form bigger structures. If such coalescence took place, it implies that not all threads with mean diameter ${D}_{F}$ were effectively charged, because otherwise they would repel due to electrostatic repulsion. A possible reason may be the high flow rates used in the operation of the atomizer. Thus, the procedure obviously has room for improvement and optimization.

**Figure 9:**
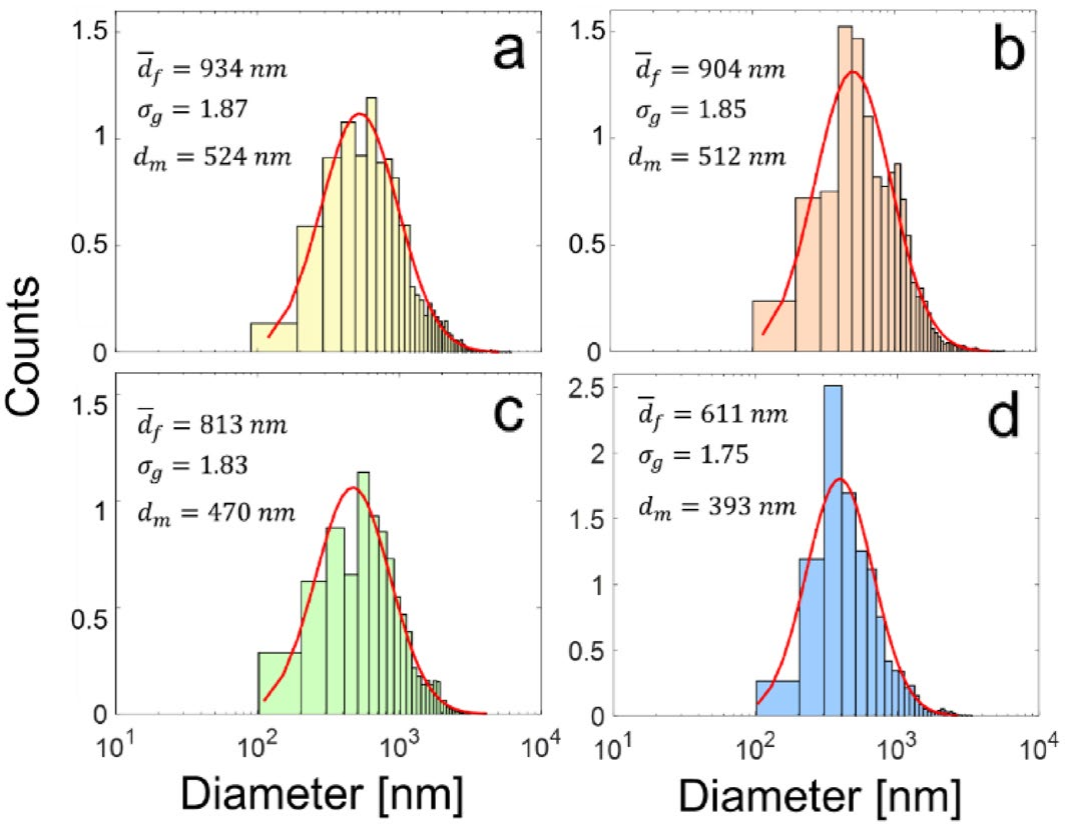
Fiber diameter distributions for varying liquid flow rates: (**a**) $2.04 \; \mathrm{ mL}/\mathrm{h}$, (**b**) $2.40 \; \mathrm{mL}/\mathrm{h}$, (**c**) $6.60\; \mathrm{mL}/\mathrm{h}$, and (**d**) $11.7 \; \mathrm{mL}/\mathrm{h}$.

Furthermore, the fiber diameter distributions obtained by processing microscopy images with the in-house developed software are presented in Fig. 9. These fibers exhibited polydisperse diameter distributions, as indicated by the values of the geometric standard deviation (${\sigma }_{g}>1.4)$, and can be well described by a log-normal fit. Here, it is important to note that during image processing only fibers were considered. That is, relics of relatively large droplets such as spherical or quasi-spherical particles as well as any other solid structures lacking a one-dimensional shape were disregarded aiming at focusing on the description of the fibrous material only. In addition, the number of spherical-to-fibrous geometries observed in the resulting material suggests that, at least qualitatively, there seems to be an increase of the number of spherical particles with increasing liquid flow rate, which seems consistent with the residence time of the liquid in the mixing region of the nebulizer and the efficiency of the mixing process. The distributions reveal the geometric mean diameters (${\overline{d}}_{f}$) of the fibers ranging from 600 to 930 nm, showing a gradual decrease with increasing liquid flow rate. The relationship between ${\overline{d}}_{f}$ and ${Q}_{l}$ follows a polynomial expression $\overline{d}_{f} = - 1.52Q_{l}^{2} - 11.4Q_{l} + 952$ (${Q}_{l}$ in mL/h). It is worth mentioning that the mode diameters (${d}_{m}$) of all distributions are smaller than the geometric mean values. For example, a ${d}_{m}$ of 393 nm was obtained at the highest flow rate.

**Figure 10:**
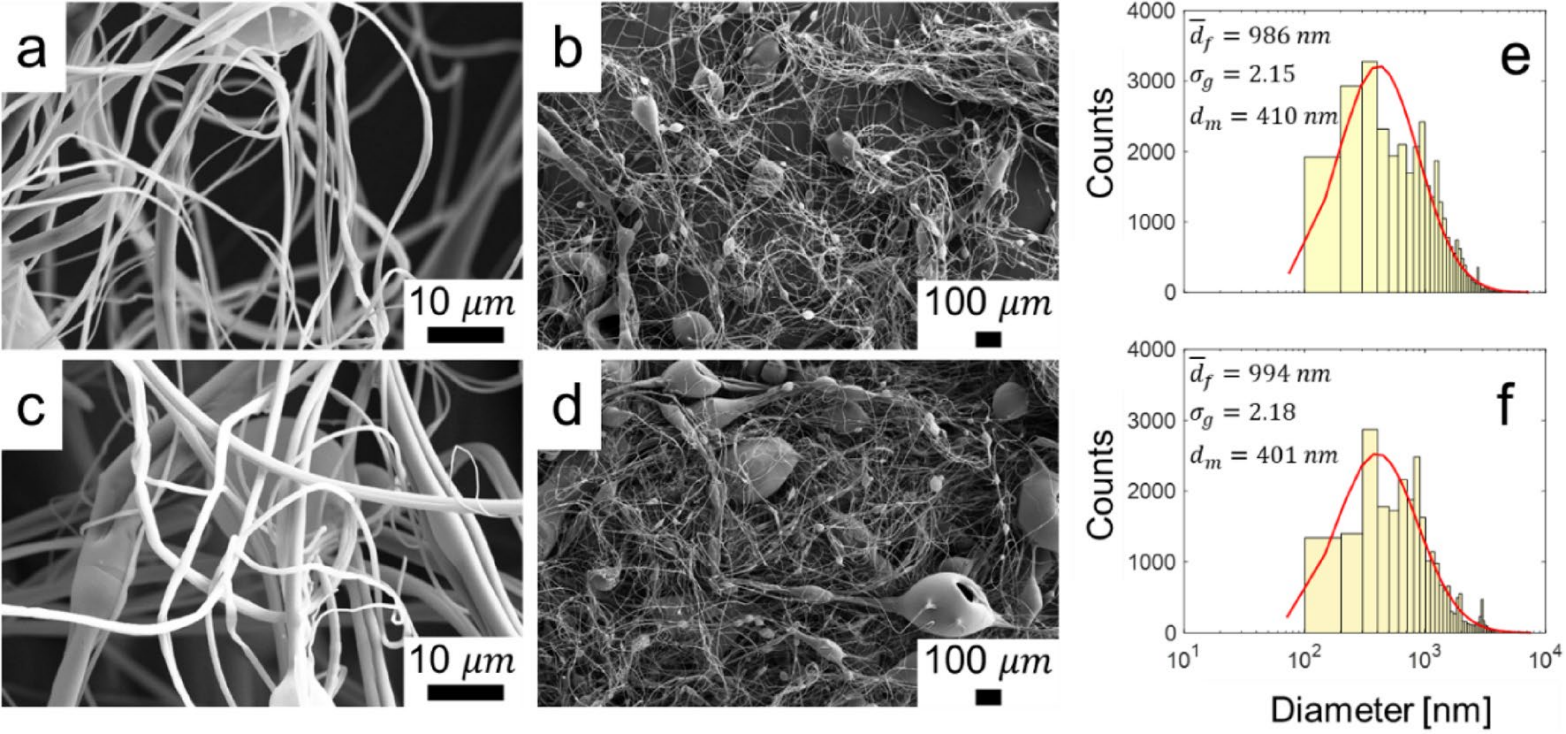
SEM images of PVP fibers containing (**a**,**b**) CNTs and (**c**,**d**) GOSs. Size distribution of PVP fibers with (**e**) CNTs and (**f**) GOSs.

Additionally, the system was used to process solutions containing CNTs and GOSs, which resulted in the formation of fiber mats composed of randomly oriented microfibers, as shown in the SEM images of Fig. 10. In these particular cases, external heat was required and provided through a heat gun, to fully evaporate the solvent and form the fibers. It is likely that addition of relatively low amount of carbon-based nanomaterial modified the physicochemical properties of the solutions because under similar atomization conditions as those of PVP they did not form fibers unless the external heat was applied. The effect of addition of carbonaceous nanomaterials has been reported with diverging results. For instance, Ramazani and Karimi reported that addition of GO to poly($\varepsilon$-caprolactane) solutions caused a decrease in the dynamic viscosity with increasing GO content^54^. Kim and Kim reported that despite increasing the content of multi-walled CNTs (MWCNTs) in PVP (average ${M}_{w}=29{,}000$ g/mol) solutions did not change the dynamic viscosity as a function of shear rate (up to 100 s^−1^) but they observed an increase of the solutions’ apparent extensional viscosity^55^. Conversely, Shang et al.^56^ reported that addition of MWCNTs up to a concentration of 5 wt% to isopropanol-ethyl acetate-based PVP (average ${M}_{w}=1{,}300{,}000$ g/mol, as used by us) solutions resulted in an increase of their zero-shear viscosity but they exhibited a shear thing behavior up to 300 s^−1^. It is thus clear that detailed studies are needed to elucidate the effect of the CNTs and GOSs on PVP solutions. Nevertheless, the scope of this work consists of demonstrating the feasibility of incorporating these materials into the fibers. The diameter distribution of the fibers obtained herein follows a lognormal pattern with a relatively high degree of polydispersity, as depicted in Fig. 10e and f. The geometric mean diameter of these fibers is approximately 990 nm.

**Figure 11:**
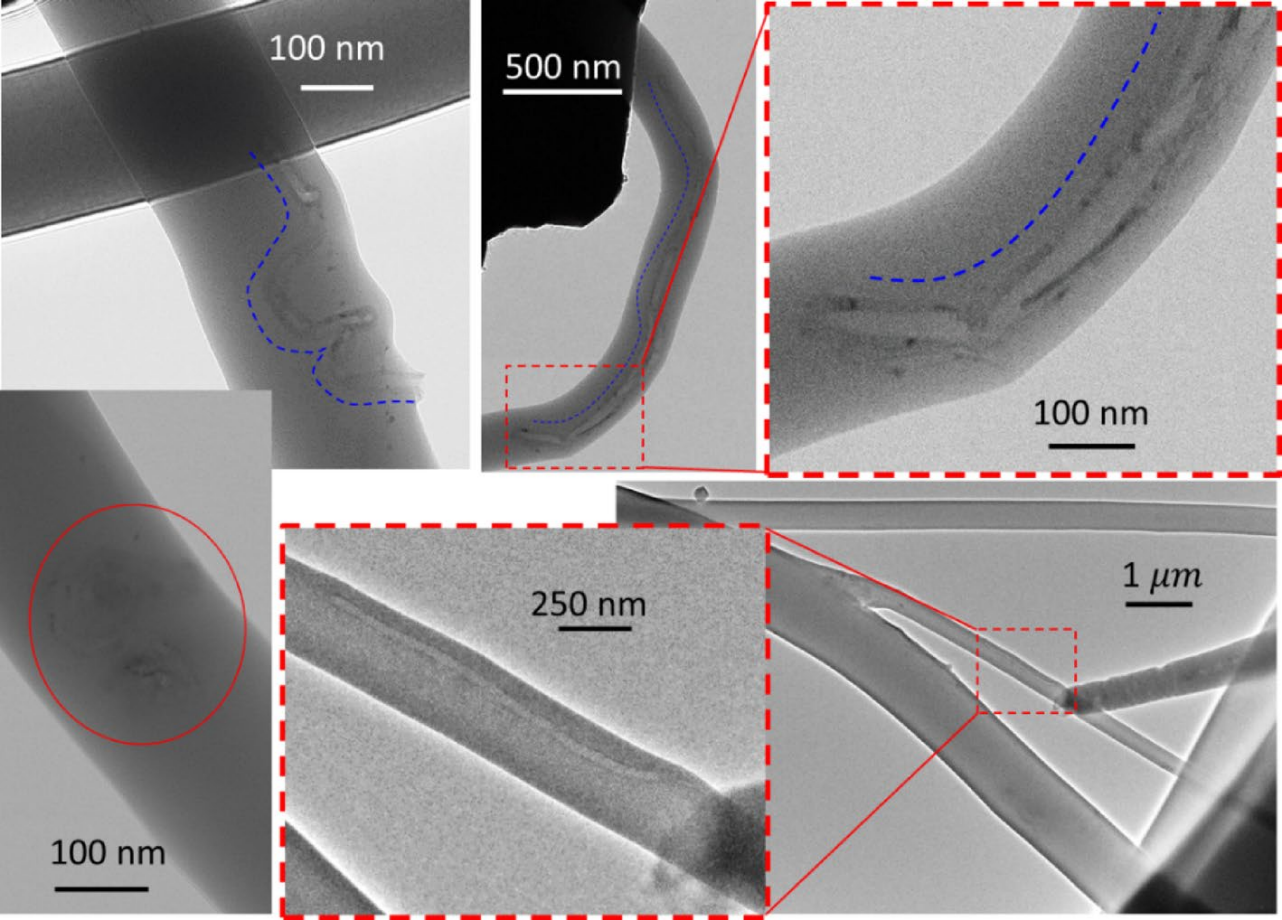
TEM images of PVP fibers containing CNTs. Blue, dotted lines to guide the eye only.

**Figure 12:**
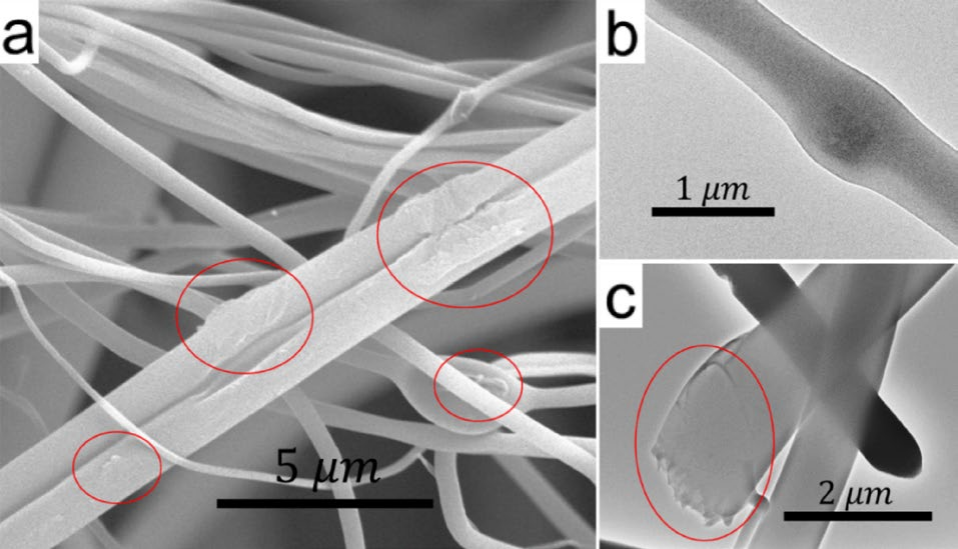
Microscopy images of GO-containing PVP fibers. (**a**) SEM, (**b**) and (**c**) TEM. The red ovals indicate GO sheets.

Both CNTs and GOSs pose challenges when it comes to their observation using microscopy tools, as they are embedded within the carbonaceous polymer matrix. However, a thorough examination of CNT-containing fibers using TEM revealed that the nanotubes appear to be embedded in the polymer matrix and aligned along the main axis of the fibers, as shown in Fig. 11. Conversely, the observation of GOSs was more intricate than that of CNTs. Nonetheless, Fig. 12 displays TEM and SEM images that seem to depict GOSs embedded in the PVP matrix. These structures could potentially enhance the strength of polymer composites. Despite further investigations are required to determine the optimal composition these promising findings encourage the continued advancement of this technique.

## Conclusions

A highly efficient method with the potential for industrial scalability was presented for synthesizing polymer fibers and composite fibers. The approach involved ejecting multiple liquid filaments derived from a bulk solution, which were fragmented through a turbulent, micro-mixing motion inside the atomizing device (*Flow Blurring* process). This method produced liquid threads with mean diameters ranging from 25 to 45 µm. The as-ejected filaments were then charged in-flight through interaction with ionized air flows having a selected turbulent signature, and subsequently stretched using electric fields. Solutions of PVP, as well as PVP containing CNTs or GOSs, were successfully synthesized into microfibers using this method. The geometric mean diameters of the synthesized fibrous materials ranged from 100 to 900 nm, achieved through liquid flow rates of up to 0.2 mL/min. The proposed synthesis method is robust, yet relatively simple, and its innovative nature allows for potential adjustments and optimization. Importantly, this method shows promise for scalability and potential application in industrial systems.

## Data Availability

The datasets used and/or analysed during the current study are available from the corresponding author on reasonable request.
