# Author Correction: The high-throughput atomization of polymer solutions for fiber synthesis in a single step aided with corona ionizers

**DOI:** 10.1038/s41598-023-42867-8

**Published:** 2023-09-22

**Authors:** Luis B. Modesto-López, Alfonso M. Gañán-Calvo

**Affiliations:** 1 Departamento de Ingeniería Aeroespacial y Mecánica de Fluidos, ETSI, Universidad de Sevilla, Camino de los Descubrimientos S/N, 41092 Seville, Spain; 2 ENGREEN, Laboratory of Engineering for Energy and Environmental Sustainability, Universidad de Sevilla, 41092 Seville, Spain

Correction to: *Scientific Reports* 10.1038/s41598-023-39801-3, published online 03 August 2023

The original version of this Article contained an error in the Acknowledgments section.

“We acknowledge financial support from the Government of Spain through a RETOS grant (PID2019-108278RB-C31). LBML acknowledges financial support from Junta de Andalucía, Spain, through PAIDI (P18-FR-3623) and FEDER (US-1380775) grants.”

now reads:

“We acknowledge financial support from project PID2019-108278RB-C31 financed by MCIN/AEI/10.13039/501100011033. LBML acknowledges support from Junta de Andalucía, Spain, through PAIDI (P18-FR-3623) and FEDER (US-1380775) grants.”

The original Article has been corrected.

